# Physical Exercise-Induced Myokines in Neurodegenerative Diseases

**DOI:** 10.3390/ijms22115795

**Published:** 2021-05-28

**Authors:** Banseok Lee, Myeongcheol Shin, Youngjae Park, So-Yoon Won, Kyoung Sang Cho

**Affiliations:** 1 Department of Biological Sciences, Konkuk University, Seoul 05029, Korea; jitcouk@konkuk.ac.kr (B.L.); shmych1022@konkuk.ac.kr (M.S.); pyj7377@konkuk.ac.kr (Y.P.); 2 Korea Hemp Institute, Konkuk University, Seoul 05029, Korea

**Keywords:** Alzheimer’s disease, amyotrophic lateral sclerosis, exercise, Huntington’s disease, muscle–brain axis, myokines, neurodegenerative diseases, Parkinson’s disease

## Abstract

Neurodegenerative diseases (NDs), such as Alzheimer’s disease (AD), Parkinson’s disease (PD), Huntington’s disease (HD), and amyotrophic lateral sclerosis (ALS), are disorders characterized by progressive degeneration of the nervous system. Currently, there is no disease-modifying treatments for most NDs. Meanwhile, numerous studies conducted on human and animal models over the past decades have showed that exercises had beneficial effects on NDs. Inter-tissue communication by myokine, a peptide produced and secreted by skeletal muscles during exercise, is thought to be an important underlying mechanism for the advantages. Here, we reviewed studies about the effects of myokines regulated by exercise on NDs and their mechanisms. Myokines could exert beneficial effects on NDs through a variety of regulatory mechanisms, including cell survival, neurogenesis, neuroinflammation, proteostasis, oxidative stress, and protein modification. Studies on exercise-induced myokines are expected to provide a novel strategy for treating NDs, for which there are no adequate treatments nowadays. To date, only a few myokines have been investigated for their effects on NDs and studies on mechanisms involved in them are in their infancy. Therefore, future studies are needed to discover more myokines and test their effects on NDs.

## 1. Introduction

Neurodegenerative diseases (NDs) such as Alzheimer’s disease (AD), Parkinson’s disease (PD), Huntington’s disease (HD), and amyotrophic lateral sclerosis (ALS) are characterized by progressive loss of neurons and accumulation of abnormal protein aggregates [1]. They are accompanied by cognitive impairments, memory loss, and locomotor deficits and share several fundamental processes, including cell survival, neurogenesis, neuroinflammation, proteostasis, oxidative stress, and protein modification [2,3,4,5,6,7]. The pathological hallmark of each ND contains an abnormal aggregation of different proteins: amyloid-β (Aβ) and tau in AD, α-synuclein in PD, huntingtin in HD, and several proteins including Tar DNA-binding protein of 43 kDa (TDP-43) and mutant superoxide dismutase 1 (SOD1) in ALS [8]. Pathological deposition of these proteins in neurons takes place long before psychological symptoms of each ND appear, and substances that can block their abnormal aggregation are expected to be used as preventive or therapeutic agents for NDs [9,10,11,12,13].

Results of numerous studies conducted on human and animal models over the past decades have shown that exercise has beneficial effects not only on physical health, but also on neuronal functions, resulting in improved learning and memory, inhibition of neurodegeneration, and reduction of depression [14]. Exercise can increase brain volume or connectivity by enhancing neurogenesis and synaptic plasticity and changing metabolism and vascular function [14,15]. Beneficial effects of exercise on brain health are thought to be mediated by several factors, including increased trophic factors such as brain-derived neurotrophic factor (BDNF), changed expression levels of many genes, decreased inflammation, and improved brain redox status [16,17,18,19,20]. 

Since exercise has a beneficial effect on brain function, it is thought to be able to slow the onset or progression of various NDs. In some studies, regular physical activity has less side effects, but better effects than currently used treatments for NDs [21,22,23]. The effect of exercise on NDs has been mostly studied on AD, one of the NDs with the highest incidence. Many systematic reviews and meta-analyses have shown that physical inactivity is one of the most common risk factors for AD development and that physically active elderly people have a lower risk of AD and dementia [23,24]. Moreover, beneficial effects of exercise on brain function and cognitive behavior have been repeatedly confirmed in AD mouse models [25]. In addition, physical exercise improved the physical functioning of people with PD [26] and it has been demonstrated that exercise protected the rodent models of PD from neurodegeneration [27,28,29,30,31]. Furthermore, studies on other NDs such as HD and ALS have also reported that exercise has a beneficial effect on these diseases [32,33]. However, some studies have failed to confirm the effect of exercise on NDs, and other studies have even shown that exercise has a rather harmful effect on NDs [34,35,36]. In addition, few studies have accurately proven the effect of exercise on NDs using appropriate biomarkers. Thus, verifying exercise’s effect through high-quality studies with large sample numbers is required in the future [32,35]. Nevertheless, it is clear that exercise is worth considering as an important therapeutic strategy if there are accurate data about the effect of exercise on each disease according to its type and amount.

Given the beneficial effect of exercise on neuronal health, various recent studies have revealed the importance of the muscle–brain axis in transmitting the effect of exercise to the brain as well as the role of muscles in secreting various factors for regulating brain function [37]. In this review, we examine the definition, function, and regulation of myokines released from skeletal muscles during exercise and their effects on neuronal health. In addition, molecular mechanisms underlying myokine’s effects on NDs are investigated. Finally, the potential of myokine as a therapeutic agent for NDs and the direction of future myokine research are discussed.

## 2. Myokines and Neuronal Health

Molecular mechanisms of how physical activity exerts protective effects against NDs have not been completely elucidated yet. However, inter-tissue communication by myokine is being proposed as a strong candidate for them [38]. Myokine was first postulated by Pederson in 2003 [39]. As a cytokine or a peptide produced and secreted by skeletal muscles, it exerts autocrine, paracrine, and endocrine effects [37,40]. It mediates crosstalk between muscles and other organs, promoting neurogenesis and vascularization in the brain [41,42], inducing browning of white adipose tissue (WAT) [43], accelerating hepatic glucose production, and stimulating insulin secretion by pancreatic β-cells [44,45]. In the following, we will review studies on factors secreted by muscles during exercise, which are known to affect neuronal health.

### 2.1. Apelin

Apelin, named APJ endogenous ligand, was first isolated in 1998 as a ligand for orphan G protein-coupled receptor APJ [46]. Apelin and APJ are widely distributed in the body and play an important role in cell protection in many organs [47]. The gene APLN encodes the pre-proprotein of apelin. Active apelin-13, pyroglutamate-apelin-13, apelin-17, and so on are produced by post-translational modification of the pre-proprotein [48].

Apelin was discovered as an exercise-induced myokine with an increased expression level after performing an 8-week endurance training program in 11 obese non-diabetic male subjects, and was confirmed to be secreted by human primary myotubes in vitro [49]. In addition, apelin level was decreased in an age-dependent manner in humans and rodents, and muscle function was decreased with aging in mice deficient in either apelin or its receptor APJ [50].

It is well-known that apelin contributes to conditions such as cardiovascular disease, obesity, and cancer [51]. In addition, apelins are distributed throughout the nervous system and have been reported to possess neuroprotective effects [52,53,54,55,56,57,58]. The role of apelin in NDs has only been studied recently. The results from these studies, which have been mainly focused on AD and PD using rodent models or cellular models, have suggested that apelin has beneficial effects on these diseases through various pathways [59,60,61,62,63,64,65,66,67]. One study has shown that apelin deficiency can accelerate ALS-like phenotype in an SOD1 (G93A) mouse model [68]. However, studies on changes in the expression level of apelin and its effects in human ND patients are insufficient. It is necessary to confirm the relationship of apelin with ND and its potential as a therapeutic agent through human studies in the future.

### 2.2. BDNF

BDNF is a member of the neurotrophin family, which regulates neural circuit development and function [69]. It was purified from porcine brain in 1982 as a neuronal survival factor [70]. Subsequently, numerous studies have shown that BDNF can perform a variety of functions. BDNF has been known to penetrate the blood–brain barrier (BBB) by a saturable transport system [71]. It can promote the survival and growth of a variety of neurons, affect synaptic transmission, enhance neurogenesis, and alter activity-dependent synaptic plasticity [72]. BDNF is initially synthesized as pre-pro-peptide that is cleaved to pro-BDNF, then the pro-BDNF is converted to mature BDNF by furin endopeptidase intracellularly, or by proteases such as plasmin or MMP7 extracellularly [73]. Mature BDNF is involved in neuronal plasticity such as neurogenesis, neurite arborization, and synaptogenesis by binding to tropomyosin receptor kinase B (TrkB) [74,75]. On the other hand, pro-BDNF functions in programmed neuronal death, neurite retraction, and synaptic pruning through a p75 neurotrophin receptor (p75NTR) [75].

According to previous studies, BDNF was increased in both acute aerobic and resistance exercises [76,77]. More recently, it has been shown that BDNF is expressed and secreted in muscles, and the muscle-derived BDNF acts as a hormone and affects whole-body metabolism and insulin secretion [78,79]. Additionally, increasing BDNF levels could mimic beneficial effects of exercise, including improving cognitive impairment and promoting combined adult hippocampal neurogenesis in 5×FAD mice [80]. In addition, TrkB inhibitor treatment blocked beneficial effects of exercise in a rat model of PD [81], suggesting that BDNF is an important factor mediating beneficial effects of exercise on ND.

Numerous studies have reported the importance of BDNF in ND pathology. In AD patients, hippocampal BDNF mRNA [82] and peripheral BDNF levels [83] were decreased compared to those in the control group, and serum BDNF levels were negatively correlated with future occurrence of dementia and AD [84], suggesting that BDNF plays a role in protecting the brain from AD. Consistently, studies using numerous in vitro and in vivo AD models have clearly shown that BDNF has neuroprotective effects [85,86,87,88,89]. In addition, genetic studies on human AD patients have reported that BDNFVal66Met SNP, known to reduce synaptic BDNF release [90], is associated with greater cognitive impairment and higher vulnerability of hippocampus-frontal connectivity to primary AD pathology [91,92]. Meanwhile, BDNF acts as a neurotrophic factor in dopaminergic neurons of the substantia nigra [93], and its neuroprotective effect has been verified in various PD models [94,95,96,97,98]. Recent studies have reported that α-synuclein, a PD-related protein, can block BDNF-TrkB signaling and induce dopaminergic cell death [99,100], suggesting that BDNF-TrkB signaling is implicated in PD pathology. The idea that degenerative diseases of the nervous system might be due to insufficient supply of neurotrophic factors has generated great interest in BDNF as a potential therapeutic agent.

### 2.3. CTSB

Cathepsin B (CTSB) is a lysosomal cysteine protease and is involved in catabolism of proteins in lysosome and autophagy [101,102]. Interestingly, it can be secreted from cells and plays a role in proteolysis of extracellular components, thus contributing to tumorigenic processes including apoptosis and invasion [103].

A recent study has demonstrated that CTSB is a myokine increased by aerobic exercise [41]. In the same study, treatment with AMP-kinase agonist induced CTSB secretion from cultured skeletal muscle cells, suggesting that CTSB expression is dependent on AMP kinase that has been suggested to mediate beneficial effects of exercise [41,104,105]. More recently, it has been reported that long-term exercise training (35  ±  15 years) significantly reduced resting serum levels of BDNF and plasma levels of CTSB in human, although they are beneficial for the brain and muscle health, which suggests that exercise may sensitize BDNF and CTSB signaling [106].

In CTSB-deficient condition, unlike in wild type (WT), beneficial effects of exercise, such as improvement of memory and adult neurogenesis, were not found, suggesting that CTSB mediates beneficial effects of exercise on brain health [41]. Furthermore, numerous studies using various models have suggested that CTSB would be beneficial to AD and PD by reducing Aβ and α-synuclein accumulation respectively, due to autophagy and lysosome-related functions of CTSB in the brain [107,108,109,110,111,112]. In the similar context, treatment with Z-Phe-Ala-diazomethylketone (PADK; also known as ZFAD), a CTSB activator, reduced Aβ deposition and improved synaptic and cognitive dysfunctions in an APP/presenilin-1 (PS1) and mild cognitive impairment (MCI) mouse model [112].

### 2.4. CX3CL1

C-X3-C Motif Chemokine Ligand 1 (CX3CL1), also called fractalkine or neurotactin, is a type of chemokines which are secreted proteins that play an important role in inflammation and trafficking of white blood cells during immune surveillance [113,114]. G-protein-coupled receptor C-X3-C Motif Chemokine Receptor 1 (CX3CR1) has been identified as the receptor for CX3CL1 and implicated in the function of leukocytes and microglia [115,116]. CX3CL1 has a cysteine signature motif, CX3C, which contains three unspecified amino acids between cysteine residues [113]. It is synthesized as a transmembrane molecule, and a chemo-attractive-soluble form containing CX3C motif is generated by metalloproteases ADAM 10 and ADAM 17 [117,118,119]. Based on its chemo-attractive role, CX3CL1 has been intensively associated with various inflammatory diseases [119]. 

CX3CL1 was identified as a protein secreted from skeletal muscles [120]. Several recent studies have found that CX3CL1 mRNA levels were increased in muscles and its protein levels were increased in plasma after acute or resistance exercise [121,122,123,124]. These findings suggest that CX3CL1 is an exercise-induced myokine that might be involved in communication between skeletal muscles and other organs.

In the brain, CX3CL1 might suppress neuroinflammation through activation of microglial CX3CR1 [125]. Given that neuroinflammation is an important factor in progressing NDs, the CX3CL1-CX3CR1 pathway is expected to have beneficial effects on NDs. However, to date, the role of the CX3CL1-CX3CR1 pathway in NDs is inconclusive and findings are controversial. First of all, there are conflicting reports about levels of CX3CL1 in AD patients. Some previous studies reported that CX3CL1 levels were decreased in brains and cerebrospinal fluid (CSF) of AD patients and in brains of AD model mice [126,127,128,129], while other studies reported that CX3CL1 was increased more in CSF or plasma of MCI and AD patients than in healthy people [130,131]. CX3CL1 expression is also inconsistent in other NDs. CX3CL1 levels in CSF of PD patients did not change compared to age-matched controls [132], whereas CX3CL1 levels in the putamen of HD patients were downregulated [133]. CX3CL1 mRNA levels in spinal cords of ALS model mice were reported to increase at 40 days of age but decrease at 90 and 120 days compared to those in WT mice [134]. In most ND-related studies, it was shown that the soluble form of CX3CL1 had beneficial effects [135,136,137,138,139,140,141], suggesting that increased CX3CL1 level in the blood after exercise might have beneficial effects on NDs. However, results of CX3CR1 deficiency are clearly contradictory. While CX3CR1 deficiency showed beneficial effects in some studies [142,143,144,145,146,147], it showed deteriorating effects in other studies [127,148,149,150,151]. The reason for these inconsistent results might be because the CX3CL1-CX3CR1 pathway is related to the function of microglia, which can play an opposing role in the ND process by acting on neuroinflammation and phagocytic clearance at the same time. To develop a therapeutic agent based on CX3CL1, in-depth studies on more detailed mechanisms should be conducted.

### 2.5. FGF2

Fibroblast growth factor 2 (FGF2), also known as basic fibroblast growth factor (bFGF) and FGF-β, is one of the growth factors that plays an essential role in neural development and proliferation of neural stem and progenitor cells [152]. FGF2 is ubiquitously expressed in various tissues, including brain and muscles, and aerobic exercise has been found to increase FGF2 expression levels in animal models [153,154,155]. However, it is unclear whether exercise can increase FGF2 expression in humans [156]. FGF2 is unconventionally secreted from cells, in which it forms lipidic membrane pores by binding to phosphoinositide PI(4,5)P2, and it is secreted by the separating action of membrane proximal heparan sulfates proteoglycans [157,158,159,160].

As a neurotrophic factor, FGF2 is known to stimulate neurogenesis and angiogenesis in adult brains and developing brains [161,162,163,164,165,166,167]. Accordingly, it exerted beneficial effects in some ND animal models [168,169]. Especially, FGF2 treatment inhibited Aβ production in primary cultured neurons of APP/PS1 mice and APPswe-HEK293 cells [170,171] and improved synaptic transduction, plasticity, and neurogenesis in an APP/PS1 mouse, while reducing hippocampal Aβ deposition and memory impairment [170]. However, effects of FGF2 on AD or ALS are more complex than expected. FGF2 levels in brains of AD patients and serum and CSF of ALS patients were reported to be elevated compared to those in normal controls [172,173,174]. FGF2 elevated the expression of tau, glycogen synthase kinase-3 (GSK-3) activity, and GSK-3-mediated tau phosphorylation [175]. Furthermore, it reduced neurogenesis in cultured neural progenitor cells derived from adult rat hippocampus [176] and induced dysregulation of dentate gyrus neurogenesis [177]. Contrary to the expectation that reduced FGF2 levels would worsen the phenotype of ALS, FGF2 deficiency significantly delayed disease onset and improved impaired motor performance in mutant SOD1 mice, a common ALS model [178].

### 2.6. FGF21

Fibroblast growth factor 21 (FGF21), a hormone belonging to the FGF superfamily, was first discovered in mouse embryos in 2000 [179]. Although this hormone is mainly expressed in the liver, it is also produced in various organs, including muscle, adipose tissue, pancreas, and heart, regulating energy homeostasis in an autocrine, paracrine, or endocrine manner [180,181]. As a result of preclinical studies, FGF21 has been attracting attention for its potential use as a treatment for metabolic syndromes such as diabetes by increasing insulin sensitivity, improving glucose tolerance, and reducing body weight [182]. Exercise elevated blood FGF21 levels mainly due to increased FGF21 expression in the liver [183,184,185]. In addition, expression levels of FGF receptor-1 (FGFR1) and β-Klotho (KLB), a co-receptor, were increased in adipose tissues during exercise, thus improving the sensitivity of adipose tissues to FGF21 [186] and promoting browning of white adipose tissues [187]. 

FGF21 was reported as a myokine regulated by the phosphoinositide 3-kinase (PI3K)/Akt axis [188], and its expression was increased in muscles during acute aerobic exercise [189]. Interestingly, FGF21 is also known as ’mitokine’, which is expressed and secreted in cells with mitochondrial damage due to autophagy dysfunction, endoplasmic reticulum stress, and mitochondrial gene abnormalities, and non-autonomously affects metabolism of other cells [190,191,192,193,194]. In particular, mitochondria damage in muscle cells induced an ATF4-dependent increase of FGF21 expression, thereby inducing systemic metabolic adaptation such as improved insulin sensitivity, increased energy expenditure, and enhanced lipid catabolism and WAT browning [190].

In addition to metabolic function, FGF21 can penetrate the BBB [195], and has been reported to improve cognitive performance in diabetes and trauma models [196,197,198,199]. In various brain damage models, FGF21 prevented inflammation and BBB disruption through PPAR-γ activation and induced neovascularization [196,197,198,200,201,202,203,204]. Although not many studies have been conducted on the effect of FGF21 on NDs, several recent preclinical studies have shown that FGF21 has a neuroprotective effect in ND models by affecting several signaling pathways. In both in vivo and in vitro AD models, FGF21 has shown anti-inflammatory and antioxidant effects, and prevented amyloid plaque formation, neurofibrillary tangle formation, and neurodegeneration [205,206,207,208]. Moreover, FGF21 treatment led to alleviated dopaminergic neuron loss, improved mitochondrial function and behavioral ability, and decreased inflammation in PD models [209,210,211,212]. Additionally, intraperitoneal injection of R1Mab1, a pharmacological agonist of FGF21 which is an IgG humanized monoclonal antibody with agonistic activity on FGFR1, improved the motility of the ALS model mice [213].

### 2.7. IGF-1

Insulin-like growth factor 1 (IGF-1), also called somatomedin C, is a secreted peptide with a structure similar to insulin and is involved in various physiological functions [214]. IGF-1 is composed of 70 amino acids with three disulfide bonds, the position of which is the same as the disulfide bond connecting A and B chains of insulin [215]. IGF-1 is a potent myoanabolic factor, which is expressed and secreted in muscle tissue, and muscle hypertrophy can increase IGF-1 expression [216]. According to previous studies, although there is some disagreement on the proportional relationship between exercise status and serum IGF-1 levels, many studies have shown that serum IGF-1 levels were increased in the elderly after aerobic and resistance exercise [217,218,219]. Moreover, a study revealed that IGF-1 is indispensable for exercise-induced neurogenesis [220].

A previous study showed that IGF-1 enters into the brain through the blood-CSF pathway [221]. Although it is known that IGF-1 plays an important role in brain development and neurogenesis and that large amounts of IGF-1 receptors are expressed in the brain [222], its role in cognitive function and NDs of the aging brain is still complex and controversial [214]. In some studies, serum IGF-1 levels in the elderly were positively correlated with cognitive function, whereas in other studies, those were not correlated or reversely correlated [223,224,225,226]. Results from studies on IGF-1 levels in ND patients are also complicated. A large-scale study on AD patients has reported that low serum IGF-1 levels are associated with an increased risk of developing AD dementia [227]. However, a meta-analysis based on results of nine studies comparing serum IGF-1 levels with normal subjects failed to find a significant difference between AD patients and normal subjects [228]. On the other hand, most studies on the association between PD and IGF-1 levels have reported higher IGF-1 levels in PD patients than in normal subjects [229]. More importantly, studies using rodent PD, HD, or ALS models have consistently shown that IGF-1 has beneficial effects on these diseases [230,231,232,233,234,235,236]. However, results from studies on the role of IGF-1 signaling in AD mouse models are not consistent with each other. In some studies, systemic infusion of IGF-1 reduced brain Aβ levels and toxicity [237,238]. However, in other studies, decreased IGF-1 signaling alleviated Aβ toxicity in AD mice [239,240,241]. In addition, a systematic review of the literature showed that it was unclear whether circulating or brain IGF-1 could reverse or slow the rate of decline in cognitive impairment in patients with dementia [242].

### 2.8. Irisin

Irisin, named after the Greek messenger goddess Iris, is a 112 amino acids-cleaved product of fibronectin type III domain-containing protein 5 (FNDC5), a type I transmembrane glycoprotein [43]. After being proteolytically cleaved from FNDC5, irisin is secreted and functions as a myokine [243]. Irisin was initially identified in skeletal muscles, but it was later found to also be expressed in a variety of tissues, including the brain [244]. Exercise elevated the expression of transcription factor PGC-1α in muscles [19], thus enhancing the expression of FNDC5 and consequently increasing the amount of irisin secreted into the blood by proteolytic cleavage of FNDC5, and irisin level was elevated in the plasma of individuals undergoing aerobic training [43,245].

Irisin has been shown to exert beneficial effects on various tissues including bone, fat, liver, and muscle [246,247,248]. One study showed that irisin could trigger cell proliferation in hippocampal cell lines [249] and the secretion of irisin during exercise enhanced the expression of BDNF and neurotrophic genes in mouse brains [250,251]. In addition, overexpression of irisin in the brain suppressed neuroplasticity defects and memory impairment in AD model mice, and intraperitoneal injection of anti-FNDC5 eliminated the beneficial effect of exercise on AD-like phenotype [252]. Moreover, co-treatment of irisin with bone marrow stem cells protected dopaminergic neurons from degeneration and apoptotic process in a MPTP-induced PD mouse model [253]. Interestingly, a study with 14 AD patients and 25 non-demented controls revealed that CSF irisin levels were positively correlated with levels of Aβ and BDNF in CSF and cognitive status of patients with AD [254]. Similarly, one study found that serum irisin levels in patients with ALS were higher than those in normal controls and that irisin levels were negatively correlated with the extent of functional and respiratory impairment in patients with ALS [255]. A study on the effect of irisin on ND is in its infancy, and many future studies are needed to reveal the potential of irisin as an ND therapeutic or diagnostic biomarker.

### 2.9. LIF

Leukemia inhibitory factor (LIF) is a member of the interleukin-6 family of cytokines with pleiotropic functions [256] and was first identified to be able to induce the differentiation of macrophages [257]. It plays an important role in promoting proliferation, differentiation, and survival of various types of cells, including neurons, myoblasts, hepatocytes, adipocytes, megakaryocyte progenitors, and myeloid cells [258]. 

LIF has also been identified as a myokine whose expression is increased by exercise in human and animal models [259,260]. It has a secretion signaling peptide that regulates its secretion from cells [261], and its secretory property was confirmed in cultured human myotubes and mouse skeletal muscles [262]. According to a study by Broholm et al., immediately after performing an aerobic exercise for 3 hours, LIF mRNA expression levels in muscles were increased up to 4 times and then gradually decreased [259]. In a more recent study, plasma LIF content was increased about 50% after a static exercise, although it was not increased after a dynamic exercise, which means that the regulation of LIF expression might differ depending on the type of exercise [263]. Meanwhile, treatment with ionomycin, a Ca^2+^ ionophore, elevated LIF mRNA and protein expression levels in human muscle cells [259], suggesting that oscillations of Ca^2+^ concentration following muscle contraction could affect LIF transcription. In addition, in cultured human myotubes, LIF was regulated by the PI3K-Akt pathway, and expression levels of JunB and c-Myc induced by LIF were also increased in skeletal muscles after a resistance exercise [264].

Since LIF is known to be able to pass through the BBB [265], it is expected that plasma LIF can affect the brain function. Expression levels of LIF and its receptor, LIFR, were increased in brains of AD and PD patients compared to healthy controls [266]. Furthermore, LIF was increased in skin samples of ALS patients [267], suggesting that LIF might be related to the pathophysiology of NDs. However, to date, research on the role of LIF in ND pathology is very limited. A recent study has shown that LIF reduced amyloid β-induced neurotoxicity in HT-22 mouse hippocampal cell lines and primary hippocampal cells through Akt/extracellular signal-regulated kinase (ERK)-mediated c-fos induction [268]. However, LIF treatment did not show a beneficial effect on the disease progression in ALS model mice [269]. Considering the effect of LIF on brain function as a neurogenesis- and inflammation-related factor, further studies are needed to determine the association of ND with LIF using various ND models.

## 3. Molecular Mechanisms Underlying Myokine Action in Neurodegenerative Diseases

**Figure 1:**
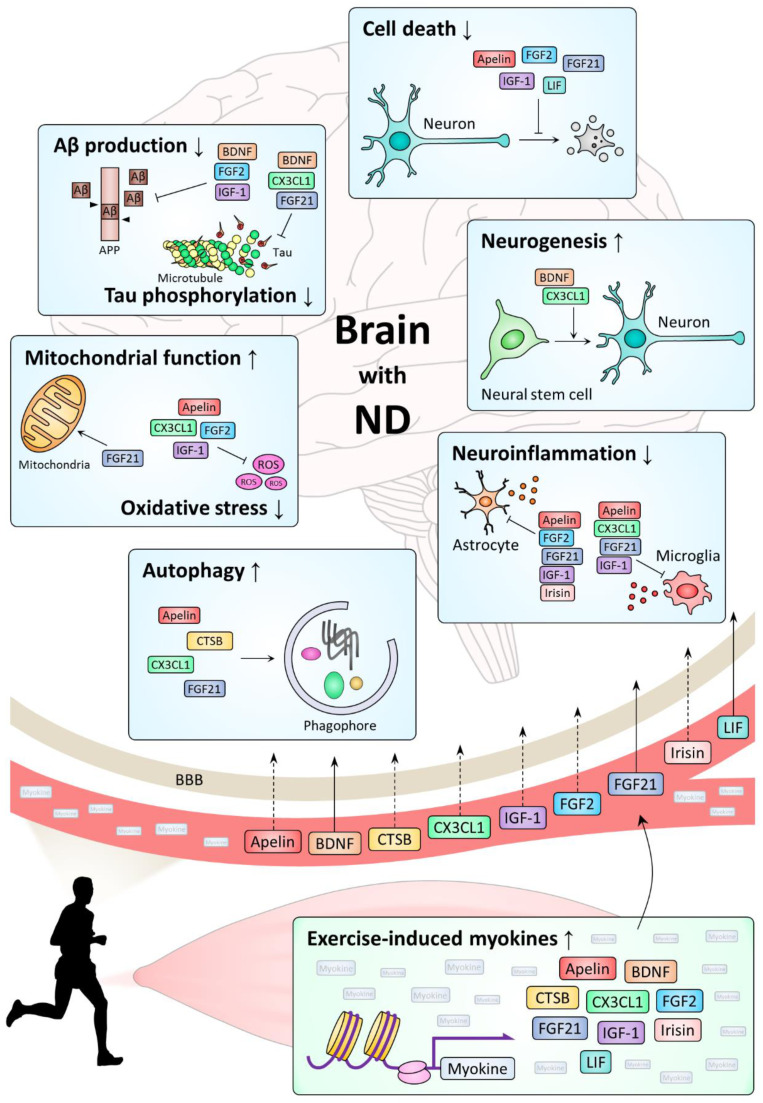
Mechanism of action of representative exercise-induced myokines in the brain with ND. While there is experimental evidence that some myokines penetrate the BBB (solid arrows from blood vessels through the BBB to the brain), others do not so far (dashed arrows from the blood vessels through the BBB to the brain).

Most NDs, including AD, PD, HD, and ALS, share pathological changes such as progressive loss of neurons, accumulation of abnormal protein aggregates, and abnormally increased neuroinflammation [1]. Studies on exercise-induced myokines have shown that myokines play a beneficial role in NDs through a variety of mechanisms, including regulations of cell survival, neurogenesis, neuroinflammation, proteostasis, oxidative stress, and protein modification (Table 1). Here, we examined actions of myokines on NDs by each mechanism (Figure 1).

### 3.1. Cell Survival

Neuronal cell death is one of the most important features in the brain of patients during ND progression [270]. During ND, neurons die by activating the death signaling pathway, resulting in brain atrophy [271]. In particular, apoptosis is an important form of cell death in ND, which includes intrinsic pathways that occur inside cells damaged by stress and extrinsic pathways that are triggered by signals from other cells [272]. In apoptosis, cell death is induced by the activation of protease activity of a series of caspases, and then, various factors activate or inhibit caspase actions to form cell death or survival signals, respectively [271]. Numerous proteins including apoptosis signal-regulating kinase 1 (ASK1), c-Jun N-terminal kinases (JNK), B-cell lymphoma 2 (BCL2)-associated X protein (Bax), and apoptotic protease activating factor 1 (Apaf-1) are involved in cell death signals that activate apoptosis of neurons, while many factors including nerve growth factor (NGF), PI3K, Akt, and BCL2 are included in neuronal survival pathways [271]. 

**Table 1:** Myokines secreted during exercise and their beneficial effects on NDs. ↑, activated or increased; ↓, inactivated or decreased.

Myokine	ND	Function	Mechanism	Model	Reference
**Apelin**	**AD**	Decreased cell death	Aβ-induced autophagy ↓ Caspase-3 activity ↓ mTOR phosphorylation ↑	Rats injected with Aβ_25–35_ and apelin-13	[62]
		Decreased cell death	BDNF/TrkB signaling pathway ↑	Rats injected with streptozotocin and apelin-13	[64]
		Anti-inflammation	Astrocyte and microglia activation ↓ IL-1β and TNF-α expression ↓		
		Decreased cell death	RIP1 and RIP3 expression ↓	Rats injected with streptozotocin and apelin-13	[67]
		Anti-inflammation	TNF-α expression ↓		
	**PD**	Decreased cell death	ERK1/2 phosphorylation ↑ ER stress ↓	SH-SY5Y cells treated with MPP^+^ and apelin-13	[60]
		Decreased cell death	PI3K signaling pathway ↑ Cytoplasmic cytochrome c ↓ Cleaved caspase-3 ↓	SH-SY5Y cells treated with 6-OHDA and apelin-13	[61]
		Increased α-synuclein clearance	PI3K/Akt/mTOR-autophagy signaling pathway ↑	SH-SY5Y cells treated with MPP^+^ and apelin-36	[65]
		Decreased cell death	IRE1α/XBP1/CHOP signaling pathway ↓	Mice injected with MPTP and apelin-13	[273]
		Increased α-synuclein clearance	Autophagy ↑		
		Increased α-synuclein clearance	AMPK/mTOR/ULK1-autophagy pathway ↑	SH-SY5Y cells treated with rotenone and apelin-13	[66]
		Decreased cell death	ASK1/JNK signaling pathway ↓	Mice injected with MPTP and apelin-36	[274]
			Caspase-3 activity ↓		
		Antioxidative stress	GSH and SOD ↑		
	**ALS**	Pro-inflammation	Microglia activation ↑	SOD1-G93A mice crossed with *apelin*^−/−^ mice	[68]
**BDNF**	**AD**	Decreased Aβ production	BACE1 and PSEN1 ↓	APPswe mice injected with TAT-BDNF peptide Rats injected with scopolamine and TAT-BDNF peptide	[275]
		Decreased tau phosphorylation	GSK3β activation ↓		
	**HD**	Increased neurogenesis	TrkB phosphorylation ↑	N171-82Q mice administered with 4′-DMA-7,8-DHF by oral gavage	[276]
**CTSB**	**AD**	Increased Aβ clearance	Proteolytic activity of CTSB itself	hAPPJ20 mice injected with Lenti-CTSB Primary cortical neurons from hAPPJ20 mice infected with Lenti-CTSB In vitro cleavage assay using Aβ_1–42_ and CTSB	[107]
		Increased Aβ clearance	Lamp1 expression ↑	APP/PS1 mice injected with AAV-CTSB	[111]
**CX3CL1**	**AD**	Pro-inflammation	IL-6 and TNF-α expression ↑	hAPPJ20 mice crossed with *CX3CR1*^−/−^ mice	[127]
		Decreased tau phosphorylation	GSK3α/β activation ↓	Tau P301L mice injected with AAV-CX3CL1	[137]
		Anti-inflammation	Microglia activation ↓		
		Pro-inflammation	NRF2/HO-1 signaling pathway ↓	*CX3CR1*^−/−^ mice injected with AAV-TAU^P301L^	[148]
		Pro-oxidative stress			
		Increased neurogenesis	TGF-β/Smad2 signaling pathway ↑	Tau P301S mice crossed with *Tg-CX3CL1* mice	[277]
		Anti-inflammation	Microglia activation ↓	APP/PS1 mice injected with MSCs carrying CX3CL1	[141]
	**PD**	Anti-inflammation	Microglia activation ↓	Rats injected with 6-OHDA and CX3CL1	[135]
		Anti-inflammation	Microglia activation ↓	*CX3CL1*^−/−^ mice injected with MPTP and CX3CL1	[136]
			TNF-α and IL-1β expression ↓		
		Pro-inflammation	Il-1β and IL-6 expression ↑	*CX3CR1*^−/−^ mice injected with AAV-α-SYN	[150]
	**ALS**	Pro-inflammation	Microglial activation ↑ IL-1β, iNOS, and TNF-α expression ↑ Arginase 1 and TGF-β expression ↓ NF-κB signaling pathway ↑	SOD1-G93A mice crossed with *CX3CR1*^−/−^ mice	[151]
		Decreased SOD1 clearance	Autophagy ↓		
**FGF2**	**AD**	Decreased cell death	Akt phosphorylation ↑	CVEC treated with Aβ_1–40_ and FGF2	[278]
		Decreased Aβ production	BACE1 expression ↓	APP23 mice injected with FGF2 N2a cells transfected with APPswe and treated with FGF2	[279]
		Anti-inflammation	iNOS expression ↓ Astrocyte activation ↓		
		Decreased Aβ production	BACE1 expression ↓	APPswe-HEK293 cells treated with GCM SH-SY5Y cells treated with FGF2	[171]
	**PD**	Antioxidative stress	GSH ↑	Primary rat embryonic mesencephalic cultures treated with 6-OHDA and FGF2	[280]
		Decreased cell death	MEK/ERK1/2 signaling pathway ↑ BAD phosphorylation ↑ AIF translocation ↓ PI3K/Akt signaling pathway ↑	SH-SY5Y cells treated with rotenone and FGF2 Primary ventral mesencephalic cultures treated with rotenone and FGF2	[281]
		Decreased cell death	Caspase-3 expression ↓	Rats injected with 6-OHDA and PEGylated FGF2	[282]
		Anti-inflammation	Astrocyte activation ↓	PC12 cells treated with 6-OHDA and PEGylated FGF2	
		Decreased cell death	MEK/ERK1/2 signaling pathway ↑ PI3K/Akt signaling pathway ↑ ER stress ↓	Rats injected with 6-OHDA and FGF2 Primary hippocampal neurons treated with 6-OHDA and FGF2	[283]
**FGF21**	**AD**	Decreased cell death	Caspase-3 activity ↓	SH-SY5Y cells treated with Aβ_1–42_ and FGF21	[206]
		Anti-inflammation	HSP90/TLR4/NF-kB signaling pathway ↓		
		Decreased cell death	Expression ratio of BCL2 to Bax (BCL2/Bax) ↑ Cleaved caspase-3 ↓	Rats injected with Aβ_25–35_ and FGF21 SH-SY5Y cells treated with Aβ_25–35_ and FGF21	[207]
		Decreased tau phosphorylation	PP2A phosphorylation ↓		
	**PD**	Increased α-synuclein clearance	SIRT1-autophagy signaling pathway ↑	Mice injected with MPTP and FGF21 SH-SY5Y cells treated with MPTP and FGF21	[210]
		Decreased cell death	Cleaved caspase-3 and JNK phosphorylation ↓ Expression ratio of BCL2 to Bax (BCL2/Bax) ↑	Mice injected with MPTP and treated with FGF21 via intranasal routine SH-SY5Y cells treated with MPP^+^ and FGF21 Primary dopaminergic neurons treated with MPP^+^ and FGF21	[211]
		Anti-inflammation	Astrocyte and microglia activation ↓ IL-1β, IL-12, IFN-γ, and TNF-α expression ↓		
		Enhanced mitochondrial function	AMPK/PGC-1α signaling pathway ↑		
	**ALS**	Anti-inflammation	Serum TNF-α, MCP-1 level ↓	SOD1-G93A mice injected with R1Mab1	[213]
**IGF-1**	**AD**	Decreased cell death	Akt phosphorylation ↑	Rats infused with Aβ_25–35_ and IGF-1 via subcutaneous osmotic minipump	[237]
		Increased Aβ clearance	Aβ carrier-mediated transport ↑	APP/PS2 mice injected with IGF-1 Choroid plexus epithelial cell culture system treated with Aβ_1–40_ and IGF-1	[238]
		Anti-inflammation	Astrocyte activation ↓		
		Decreased cell death	Mitochondrial membrane potential ↑ Cytoplasmic cytochrome c ↓ Cleaved caspase-3 ↓ Expression ratio of BCL-XL to Bax (BCL-XL/Bax) ↑	SH-SY5Y cells treated with Aβ_25–35_ and IGF-1	[284]
		Antioxidative stress	SOD and CAT activity ↑ PI3K/Akt/Nrf2/HO-1 signaling pathway ↑		
		Decreased cell death	C-myb expression ↑	SH-SY5Y cells treated with Aβ_25–35_ and IGF-1	[285]
		Decreased tau phosphorylation	p25 protein production ↓ μ-Calpain expression ↓		
		Decreased Aβ production	ADAM10 exprssion ↑ BACE1 expression ↓	APP/PS1 mice injected with IGF-1	[286]
	**PD**	Decreased cell death	PI3K/Akt signaling pathway ↑	Rats injected with 6-OHDA and IGF-1	[232]
		Decreased cell death	Caspase-3 expression and activity ↓ PARP cleavage ↓	PC12 cells treated with 6-OHDA and IGF-1	[287]
		Antioxidative stress	NRF2/HO-1 signaling pathway ↑		
		Decreased cell death	ERK1/2/CREB signaling pathway ↑ Akt/GSK3α/β/β-catenin signaling pathway ↑	Rats injected with 6-OHDA and IGF-1	[288]
	**HD**	Decreased cell death	PI3K/Akt signaling pathway ↑ Huntingtin phosphorylation ↑	Primary striatal neurons transfected with mutant huntingtin and treated with IGF-1 SH-SY5Y cells treated with IGF-1 HEK293T cells transfected with mutant huntingtin	[289]
	**ALS**	Decreased cell death	Akt/caspase-9/caspase-3 signaling pathway ↓	SOD1-G93A mice injected with AAV-IGF-1	[230]
		Anti-inflammation	Astrocyte activation ↓		
		Anti-inflammation	Astrocyte activation ↓ TNF-α expression ↓	SOD1-G93A mice crossed with MLC/mIgf-1 transgenic mice	[233]
		Decreased cell death	Cleaved caspase-9 ↓	SOD1-G93A mice injected with AAV-IGF-1 SOD1-G93A astrocyte-neuron coculture system treated with IGF-1	[290]
		Anti-inflammation	Astrocyte and microglia activation ↓ NOS activity and peroxynitrite formation ↓		
		Anti-inflammation	Macrophage invasion ↓ TNF-α expression ↓	SOD1-G93A mice injected with AAV-IGF-1	[291]
		Decreased cell death	JNK and p38 MAPK phosphorylation ↓ Bax expression ↓ BCL-2 expression ↑ Cleaved caspase-3 and cleaved caspase-9 ↓	SOD1-G93A mice injected with AAV-IGF-1	[292]
		Anti-inflammation	Astrocyte and microglia activation ↓		
**Irisin**	**AD**	Anti-inflammation	IL-1β and IL-6 level ↓ Akt/IκBα/NF-κB/COX-2 signaling pathway ↓	Primary hippocampal astrocytes treated with Aβ_25–35_ and irisin	[293]
**LIF**	**AD**	Decreased cell death	Aβ-induced autophagy ↓	HT-22 mouse hippocampal cells treated with Aβ_1–42_ and LIF	[268]

IGF-1, a well-established activator of the PI3K-Akt pathway, has been reported to inhibit cell death by increasing Akt phosphorylation in various models [236,285,288]. Interestingly, in a human HD cell model, Akt activated by IGF-1 inhibited the toxicity of huntingtin by phosphorylating it [289]. Furthermore, IGF-1 treatment inhibited apoptosis in ND models by inactivating the apoptosis pathway, such as increasing the activity of NF-kB, expression of BCL2, or inhibiting caspase activity [230,284,287,290,292,294,295]. It was also found that FGF2 and FGF21 increased Akt phosphorylation and BCL2 expression in various ND models, while inhibiting apoptosis by lowering JNK and caspase activities [207,211,278,281,282,283]. BDNF and LIF could also reduce neuronal cell death in AD models by activating the PI3K-Akt pathway [268,296]. In addition, intranigral injection of apelin-36 in an MPTP-induced PD mouse model reduced cell death by inhibiting the ASK1/JNK/caspase-3 pathway [274].

### 3.2. Neurogenesis

Adult neurogenesis occurs actively in healthy brain subjects, but falls sharply in AD brains [7], suggesting that neurogenesis is deeply associated with the onset of ND [297]. Interestingly, exercise is known to promote adult neurogenesis [298], and as neurotrophins, some myokines are believed to have beneficial effects on NDs by mediating the promoting effect of exercise on neurogenesis. In particular, BDNF and CX3CL1 overexpression induced neurogenesis in AD model mice [277,299] and enhanced AD therapeutic effects of engrafted stem cells [141,300]. Moreover, neurogenesis was increased in an N171-82Q HD mouse model after they were treated with BDNF receptor agonists [276], and some myokines, including irisin, CTSB, and apelin-13, are thought to contribute to neurogenesis by increasing the expression of BDNF in brains of animal models [41,64,250]. In addition, FGF2 stimulated neurogenesis in various models of NDs including AD, PD and HD, although high concentrations of FGF2 rather inhibited neurogenesis [168,169,170,176,177]. Meanwhile, in mutant mice with low levels of serum IGF-I, adult hippocampal neurogenesis was lowered without showing a decrease in anxiety behavior by exercise, suggesting that IGF-1 is involved in exercise-induced neurogenesis [220]. 

### 3.3. Neuroinflammation

Inflammation is a host defense system by activating innate immune cells such as microglia against infection, tissue injury, and cellular insults, and various cytokines secreted from immune cells mediate or inhibit inflammation [301]. However, chronically activated neuroinflammation in the nervous system of ND patients plays a causal role in the pathogenesis of ND [302,303]. Various clinical and preclinical studies have reported that exercise reduces neuroinflammation by increasing the expression of anti-inflammatory cytokines and lowering levels of pro-inflammatory cytokines and activated microglia [304,305]. When apelin-13 was injected intracerebroventricularly, it inhibited the activation of microglia and astrocytes and reduced the expression of IL-1β and TNF-α in a streptozotocin-induced rat model of AD [64]. This effect of apelin-13 was inhibited by treatment with K252a, a TrkB antagonist, suggesting that apelin-13 inhibits neuroinflammation through the BDNF-TrkB pathway [64]. The anti-neuroinflammation function is best known in CX3CL1. As an inhibitor of microglial activation, CX3CL1 has been proven to be able to inhibit neuroinflammation in various models for ND [127,136,141,148,150,151]. Likewise, irisin showed an anti-inflammatory effect by reducing the secretion of cytokines, IL-6 and IL-1β, from cultured astrocytes, and irisin-pretreated astrocytes protected neurons from Aβ toxicity [293]. In addition, FGF21 exerted neuroprotective effects by suppressing the expression of NF-κB in Aβ42-treated SH-SY5Y cells and pro-inflammatory cytokines in the models of PD and ALS [206,211,213]. Furthermore, a series of studies showed that IGF-1 reduced the expression of TNF-α and the activity of astrocyte and microglia in an APP/PS2 AD mouse model and a SOD1(G93A) mouse model [230,233,238,290,291,292].

### 3.4. Proteostasis

The pathology of most NDs involves pathogenic protein aggregation and deposition. Aggregation and deposition of Aβ and tau in AD, α-synuclein in PD, and huntingtin in HD are examples. Neurons and glial cells internalize these proteins into cells through endocytosis and then degrade them using autophagy-lysosome and ubiquitin-proteasome systems, thereby protecting neuronal cells [306]. Exercise activates autophagy in the brain as well as muscles [307], and it has been reported that physical activity not only lowers levels of pathogenic protein aggregation in various ND models [308,309], but also tau in normal brains of the elderly [310]. However, there are insufficient data supporting these findings, and the specific mechanism is also unclear. Nevertheless, there are reports about the effect of some myokines in the maintenance of proteostasis. Intranigral apelin-13 injection promoted α-synuclein clearance by activating autophagy in MPTP-induced PD model mice [273]. In vitro and in vivo studies have demonstrated that CX3CR1-deficient microglia have poor phagocytosis ability against tau and α-synuclein proteins [146,149]. Moreover, CX3CR1 deficiency exacerbated SOD1 aggregation and impaired the autophagy-lysosome degradation pathway in the SOD1(G93A) ALS mouse model [151]. CTSB has been identified as a regulator of autophagy and lysosomal dynamics [102,311]. Accordingly, a series of studies have shown that CTSB plays an important role in the clearance of Aβ and α-synuclein [107,108,109,111,112]. Similarly, in the MPTP mouse model of PD, FGF21 promoted autophagic degradation of α-synuclein via SIRT1 [210].

### 3.5. Mitochondrial Function and Oxidative Damage

Oxidative stress due to increased mitochondrial damage and reactive oxygen stress (ROS) plays an important role in the pathophysiology of ND [312]. Therefore, one of the possible mechanisms by which myokine exerts beneficial effects on NDs is to act as an antioxidant scavenging ROS or to protect mitochondria in the ND brain. In fact, it has been shown that some myokines have antioxidant or mitochondrial protective functions in several ND models. Most importantly, as a mitokine that is induced by mitochondrial dysfunction, FGF21 is intensively related to oxidative stress [313]. FGF21 treatment reduced oxidative stress in an Aβ-injected rat model and an Aβ-treated cell model by inhibiting HSP90-TLR4-NF-κB or PP2A-MAPKs-HIF-1α pathways [206,207]. Furthermore, FGF21 treatment not only enhanced mitochondrial functions through PGC-1α activation in human dopamine neurons [209], but also showed neuroprotective effects by stimulating the AMPK/PGC-1α axis to promote mitochondrial functions in MPTP-treated PD models [211]. Apelin-13 and FGF2 also showed neuroprotective effects in cellular PD models treated with 6-OHDA, an oxidative injury inducer [61,280]. In addition, IGF-1 promoted the survival of rat primary neurons and hypothalamic rat GT1-7 cells treated with hydrogen peroxide by the PI3K-NF-κB pathway [294]. 

### 3.6. Aβ Production and Tau Phosphorylation 

Aβ production through APP processing and hyperphosphorylation of tau are important in the pathophysiology of AD [314]. The amyloidogenic process that produces Aβ is catalyzed by beta- and gamma-secretase and takes place in the intracellular endosome [315]. It has been reported that intracranial injection of BDNF into the hippocampus reduced Aβ production in the brain of wild-type mice by upregulating gene expression of sorting protein-related receptor with A-type repeats (SORLA), which acts as a sorting receptor for APP and downregulates its processing into Aβ [316]. In addition, in vitro and in vivo studies have demonstrated that FGF2 reduced Aβ production in part by lowering BACE1 expression level [171,279]. Meanwhile, IGF-1 treatment increased α-secretase activity in a PI3K-dependent manner, and subcutaneous injection of IGF-1 increased the expression of ADAM 10 and decreased the expression of BACE1 in the cortex of APP/PS1 mice, which suppressed Aβ production by precluding the amyloidogenic pathway [286,317]. However, other studies showed that neuronal insulin receptor deficiency rather reduces Aβ deposition in APPswe mice [318]. Thus, further studies on detailed functions of IGF-1 in APP processing are needed.

Neurofibrillary tangles composed of highly phosphorylated tau proteins is a characteristic pathological feature of AD brain [319]. It is known that insoluble tau present in AD brain is phosphorylated at more than 45 residues by various kinases, including glycogen synthase kinase-3 (GSK-3), cyclin-dependent kinase-5 (cdk5), casein kinase 1 (CK1), and cyclic AMP-dependent protein kinase (PKA), while it is dephosphorylated by phosphatases such as PP2A [319]. Interestingly, BDNF stimulation of neuronally differentiated mouse embryonic cells resulted in a rapid decrease in tau phosphorylation in a PI3K-GSK-3β-dependent manner [320]. Given that the PI3K-Akt pathway negatively regulates GSK-3β [321], the BDNF-TrkB pathway might activate PI3K to inhibit GSK-3β, thereby lowering tau phosphorylation. Moreover, intraperitoneal injection of mature BDNF reduced Aβ and tau pathologies by inhibiting GSK-3β in the hippocampi of APPswe mice [275]. Similarly, when mesenchymal stem cells carrying CX3CL1 and Wnt3a were transplanted into brains of APP/PS1 mice, CX3CL1 activated PI3K-Akt signaling to inhibit GSK-3β [141]. In addition, tau phosphorylation was decreased in the hippocampi of APP23 transgenic mice when FGF2 was injected subcutaneously, but some studies showed that FGF2 rather elevates tau expression and phosphorylation by increasing GSK-3β activity in adult rat hippocampal progenitor cells [175,177,279]. Meanwhile, FGF21 induced by calorie restriction reduced tau phosphorylation through the mTOR axis, and tau pathology was ameliorated by FGF21 in Aβ-treated SH-SY5Y cells [205,207]. Furthermore, astrocyte-neuron lactate shuttle (ANLS) was implicated in decreased tau phosphorylation by FGF21 in a transwell co-culture system with C6 astrocytes and PC12 neurons [208].

## 4. Conclusions and Perspectives

In this review, we examined the beneficial effects of exercise-induced myokines in NDs and their molecular mechanisms. In particular, we focused on the direct mechanism of action of myokine on the brain. However, myokines not only act directly on the brain, but also affect systemic metabolism, and consequently can have beneficial effects on NDs. Exercise regulated the remodeling of adipose tissue, reducing lipid content and controlling lipid browning [322]. In addition, muscles were closely related to systemic lipid homeostasis [323,324]. Moreover, some myokines have been known to affect lipid homeostasis [38,325]. For example, BDNF polymorphism was associated with type 2 diabetes mellitus in Caucasian females with obesity [326], and serum BDNF level was associated with obesity in female patients with type 2 diabetes mellitus [327]. Furthermore, BDNF treatment improved the lipid metabolism of a mouse model of type 2 diabetes [328], and intracerebroventricular injection of BDNF in rats increased lipolysis in adipose tissue [329]. In addition, FGF21 was identified as a key mediator of hepatic lipid metabolism in a high-fat, low-carbohydrate ketogenic diet [330]. A recent study showed that exercise sensitizes the action of FGF21 in adipose tissues, while long-term high-fat diet-fed obese mice exhibited compromised effects of exogenous FGF21 on alleviation of hyperglycemia, hyperinsulinemia, and hyperlipidemia [186]. Meanwhile, it has been suggested that dysregulation of lipid homeostasis is associated with NDs such as AD and PD [331,332]. Several previous studies have provided evidence that diverse types of lipids influence AD pathogenesis through various mechanisms, including mitochondrial dysfunction, BBB destruction, amyloidogenesis, inflammation, and oxidative stress [331]. Although there are few definitive studies on the effects of systemic regulation of lipid homeostasis by myokine on NDs so far, considering the function of some myokines in maintaining lipid homeostasis and the importance of lipid in the pathogenesis of NDs, myokines not only act directly on the brain, but may exert beneficial effects on NDs by affecting systemic metabolism. 

Myokines may have indirect beneficial effects on NDs by affecting the composition of gut microbiota. Gut microbiota composition is affected by exercise, aging, diet, etc., and they not only affect the profile of myokines [333], but are closely related to NDs [334]. For example, according to a recent study, the gut microbiota of PD patients was different from that of normal people, which was associated with the motor phenotype [335]. Furthermore, a recent study reported that gut microbiota modulated motor deficits and neuroinflammation in a mouse model of PD [336]. Interestingly, it was reported that muscle mass decreased in germ-free mice [337], suggesting a close association between microbiota and muscle. Considering the effect of exercise on the gut microbiota, it is possible that myokine might affect the microbiota. However, few studies have been carried out on how myokines affect the composition of the microbiota. Further studies on this point are needed in the future, and the results of the studies are expected to provide more information about the role of myokine as a systemic regulator. 

Since many myokines are simultaneously expressed and secreted by the muscles during exercise, the beneficial effects of exercise are manifested not only through each myokine, but also through the interactions between them. In this respect, it is noteworthy that some myokines promote the expression of others and act on the same molecular mechanism. For example, it has been reported that apelin, IGF-1, and irisin can maximize their effects by increasing the expression of BDNF [250,338,339]. In addition, as shown in Table 1, various myokines influence NDs by modulating similar molecular pathways: for example, both FGF2 and IGF-1 activate the PI3K/AKT signaling pathway. Therefore, when their expression is increased by exercise, it can be expected that they will create synergy. Advanced studies on the interactions between them are needed, and these studies will broaden our knowledge about the benefits of exercise manifested by myokine.

Based on the results of previous studies over the past 20 years, myokine is emerging as a promising treatment for NDs. However, studies about the effects of myokines on NDs are currently in their infancy, for several reasons. First, studies to date have focused on only a few myokines. In this review, we have examined nine myokines that are increased by exercise with relatively clear evidence for their direct effects on NDs. However, they only account for a small fraction of total myokines released from skeletal muscles during exercise. In fact, previous proteomics studies have shown that muscles secrete more than 600 proteins [340]. Some myokines, including adiponectin, β-aminoisobutyric acid, bone morphogenetic protein- and retinoic acid-inducible neural-specific protein-3, ciliary neurotrophic factor, CXCL10, CXCL12, follistatin-like-1, IL-6, IL-7, IL-15, matrix metalloproteinases-2, meteorin 1, musclin, myonectin, myostatin, osteoglycin, and secreted protein acidic and rich in cysteine (SPARC), are well-known for their effects on other organs [341,342], while their effects on the nervous system remain unclear. Therefore, more myokines with effects on NDs should be newly discovered and studied in the future. Second, even for known myokines studied up to date, their efficacies for NDs have not been fully established yet. For example, as we have seen in this review, IGF-1 showed beneficial and sometimes harmful effects. This means that effects of myokines may appear differently depending on physiological conditions. To use them safely as therapeutic agents, their effects under various conditions must be accurately grasped. Third, data about detailed mechanisms of action of myokines are currently lacking. With the exception of very few myokines, the exact mechanism of action is unknown for most myokines. Moreover, despite many studies showing the beneficial effects of myokines on ND, there are few studies on the ability of myokines to penetrate the BBB. The ability to penetrate the BBB has been experimentally demonstrated only for some of the myokines, such as BDNF, FGF21, and LIF. Although there is no direct experimental evidence yet, there is no reason to rule out the possibility that the remaining myokines will penetrate the BBB. In the future, detailed studies on this issue are needed. In addition, it should be considered that different types of physical exercise can release different profiles of myokines. Since the expression level of each myokine may differ depending on the type of exercise, the effects of exercise on NDs may vary depending on it. These limitations are important obstacles that should be overcome to develop myokines as therapeutic agents.

Despite these limitations, results on beneficial effects of exercise and myokines on NDs emphasize that it is important to study myokines induced by exercise for developing treatments for NDs. Each myokines’ expression condition is diverse depending on exercise type so difference of exercise type could bring different beneficial effects by expressing different myokines. In the absence of clear treatments for most NDs, studies of myokines known to mediate beneficial effects of exercise on NDs could provide a breakthrough for the development of novel treatments for these diseases.

## Abbreviations

α-SYN	Alpha-synuclein
Aβ	Amyloid beta
AD	Alzheimer’s disease
AIF	Apoptosis-inducing factor
ALS	Amyotrophic lateral sclerosis
AMPK	AMP-activated protein kinase
APP	Amyloid beta precursor protein
ASK1	Apoptosis signal-regulating kinase 1
BACE1	Beta-site APP cleaving enzyme 1
BAD	BCL2-associated agonist of cell death
BAX	BCL2-associated X protein
BCL2	B-cell lymphoma 2
BCL-XL	B-cell lymphoma-extra large
BDNF	Brain-derived neurotrophic factor
CAT	Catalase
CHOP	C/EBP homologous protein
c-myb	Cellular myeloblastosis
COX-2	Cyclooxygenase 2
CREB	cAMP responsive element binding protein
CTSB	Cathepsin B
CVEC	Post-capillary venular endothelial cell
CX3CL1	C-X3-C motif chemokine ligand 1
CX3CR1	C-X3-C motif chemokine receptor 1
ER	Endoplasmic reticulum
ERK	Extracellular signal-regulated kinase
FGF2	Fibroblast growth factor 2
FGF21	Fibroblast growth factor 21
4′-DMA-7,8-DHF	4′-Dimethylamino-7,8- dihydroxyflavone
GCM	Glioma cell-conditioned medium
GSH	Glutathione
GSK3	Glycogen synthase kinase 3
HD	Huntington’s disease
HO-1	Heme oxygenase 1
HSP90	Heat shock protein 90
IFN-γ	Interferon gamma
IGF-1	Insulin-like growth factor 1
IκBα	Nuclear factor of kappa light polypeptide gene enhancer in B-cells inhibitor, alpha
IL-1β	Interleukin 1 beta
IL-6	Interleukin 6
IL-12	Interleukin 12
iNOS	Inducible nitric oxide synthase
IRE1α	Inositol-requiring enzyme 1 alpha
JNK	c-Jun N-terminal kinase
Lamp1	Lysosomal-associated membrane protein 1
LIF	Leukemia inhibitory factor
MAPK	Mitogen-activated protein kinase
MCP-1	Monocyte chemo-attractant protein 1
MEK	Mitogen-activated protein kinase kinase
MPP^+^	1-Methyl-4-phenylpyridinium
MPTP	1-Methyl-4-phenyl-1,2,3,6-tetrahydropyridine
MSC	Mesenchymal stem cell
mTOR	Mechanistic target of rapamycin
NF-κB	Nuclear factor kappa B
NOS	Nitric oxide synthase
NRF2	Nuclear factor erythroid 2-related factor 2
PARP	Poly (ADP-ribose) polymerase
PD	Parkinson’s disease
PGC-1α	Peroxisome proliferator-activated receptor gamma coactivator 1-alpha
PI3K	Phosphoinositide 3-kinase
PP2A	Protein phosphatase 2
PSEN1	Presenilin 1
RIP1	Receptor-interacting protein kinase 1
RIP3	Receptor-interacting protein kinase 3
SIRT1	Sirtuin 1
6-OHDA	6-hydroxydopamine
Smad2	Mothers against decapentaplegic homolog 1
SOD	Superoxide dismutase
TAT	Transactivator of transcription
TGF-β	Transforming growth factor beta
TLR4	Toll-like receptor 4
TNF-α	Tumor necrosis factor alpha
TrkB	Tropomyosin receptor kinase B
ULK1	Unc-51 like autophagy activating kinase 1
XBP1	X-box binding protein 1

## Notes

APP/PS1 mice	APP/PS1 mice overexpress human APP with Swedish mutation (K670N and M671L) and presenilin 1, bearing an L166P mutation in neurons driven by thymocyte differentiation antigen 1 (Thy-1) promoter [343].
APP/PS2 mice	APP/PS2 mice overexpress human APP with the Swedish mutation and presenilin 2, bearing an N141I mutation driven by the Thy-1 promoter and prion protein (Prnp) promoter, respectively [344,345].
APPswe mice	APPswe mice overexpress human APP with the Swedish mutation driven by the Prnp promoter [346].
APP23 mice	APP23 mice overexpress human APP with the Swedish mutation in neurons driven by the Thy-1 promoter [347].
hAPPJ20 mice	hAPPJ20 mice overexpress human APP with the Swedish mutation and Indiana mutation (V717F) in neurons driven by platelet-derived growth factor (PDGF)-β promoter [348].
MLC/mIGF-1	MLC/mIGF-1 mice express rat mIGF-1, an isoform of IGF-1, cDNA driven by skeletal muscle-specific regulatory elements from the rat myosin light chain (MLC)-1/3 locus [349].
N171-82Q mice	N171-82Q mice express an N-terminally truncated huntingtin cDNA that contains 82 glutamines and encompasses the first 171 amino acids of huntingtin (N171-82Q) driven by the Prnp promoter [350].
PS19 mice	PS19 mice overexpress human tau with a mutation (P301S) driven by the Prnp promoter [351]
R1Mab1	R1Mab1 is an IgG humanized monoclonal antibody with agonistic activity on the fibroblast growth factor receptor 1 (FGFR1) [213].
SOD-G93A mice	SOD-G93A mice overexpress human SOD1 with a mutation (G93A) and develop adult-onset motor neuron loss [352].
Tau P301L mice	Tau P301L mice overexpress human tau with a mutation (P301L) in neurons driven by the Thy-1 promoter [353].
